# A Novel *Closo*-*Ortho*-Carborane-Based Glucosamine Derivative as a Promising Agent for Boron Neutron Capture Therapy

**DOI:** 10.3390/ph18070986

**Published:** 2025-06-30

**Authors:** Daniela Imperio, Ian Postuma, Salvatore Villani, Erika Del Grosso, Laura Cansolino, Cinzia Ferrari, Silvia Fallarini, Silva Bortolussi, Luigi Panza

**Affiliations:** 1 Department for Sustainable Development and Ecological Transition, University of Eastern Piedmont, Piazza Sant’Eusebio 5, 13100 Vercelli, Italy; 2 National Institute of Nuclear Physics (INFN), Unit of Pavia, Via A. Bassi 6, 27100 Pavia, Italy; ian.postuma@pv.infn.it (I.P.); laura.cansolino@unipv.it (L.C.); cinzia.ferrari@unipv.it (C.F.); 3 Department of Pharmaceutical Sciences, University of Eastern Piedmont, Largo Guido Donegani, 2, 28100 Novara, Italy; salvatore.villani@uniupo.it (S.V.); erika.delgrosso@uniupo.it (E.D.G.); silvia.fallarini@uniupo.it (S.F.); luigi.panza@uniupo.it (L.P.); 4 Department of Clinical Surgical Sciences, Integrated Unit of Experimental Surgery, Advanced Microsurgery and Regenerative Medicine, University of Pavia, Via Ferrata 9, 27100 Pavia, Italy; 5 Department of Physics, University of Pavia, Via A. Bassi 6, 27100 Pavia, Italy

**Keywords:** autoradiography, saccharides, targeted medicine, boron, glioma cells, click chemistry, glucosamine

## Abstract

**Background:** Boron Neutron Capture Therapy (BNCT) is a promising cancer treatment that combines tumor-selective boron delivery agents with thermal neutrons to kill cancer cells while sparing normal tissue. BNCT requires boron-containing compounds that exhibit high tumor selectivity and achieve therapeutic boron concentrations within tumor cells. This work focuses on the early development of a novel boron cluster carbohydrate derivative based on the glucosamine structure. Our results indicate that this derivative may have advantages over the typical boron delivery agent used in clinical applications and may significantly improve boron delivery capacity at the cellular level. **Methods:** The performance of the compound in terms of boron uptake was tested in the U87-MG glioblastoma cell line employing neutron autoradiography imaging and quantification. **Results:** The compound was non-toxic for cells, and it showed a remarkable capacity to enrich cells with boron. The ratio between boron concentration provided in the culture medium and boron concentration achieved in cells was compared to that obtained with boronophenylalanine (BPA), the gold standard in BNCT. The result demonstrated a significantly better performance compared with BPA, showing that the novel agent can concentrate boron in cells more than in culture medium. **Conclusions**: The encouraging preliminary results provide a starting point for its potential application in in vivo tests.

## 1. Introduction

Boron Neutron Capture Cancer Therapy (BNCT) is a binary cancer treatment that exploits the nuclear reaction occurring when a ^10^B isotope captures a neutron [1].

**Figure 1:**
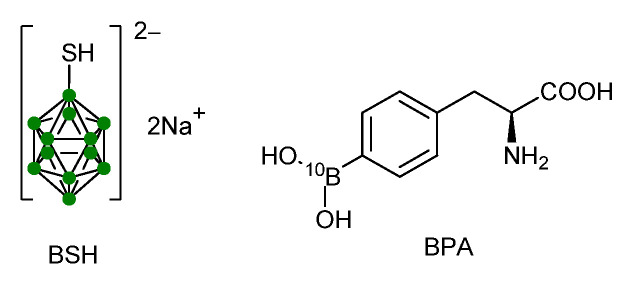
Chemical structures of mercapto-*closo*-undecahydro-dodecaborate BSH and (L)-4-dihydroxy-borylphenylalanine (BPA).

The capture reaction ^10^B(n, alpha)^7^Li generates two high-Linear Energy Transfer (LET) particles: a nucleus of ^4^He and a ^7^Li ion. In 93% of cases, lithium emerges in an excited state, which leads to de-excitation and emission of a 478 keV gamma ray. These particles have a limited range in biological tissue, approximately 8–10 microns, limiting their cytotoxic effect within the cell where the reaction occurs. The most important limitation in BNCT concerns the establishment of a favorable tumor-to-normal tissue boron ratio, characterized by high accumulation in the malignancy and negligible uptake in surrounding healthy tissues. This selective targeting is precisely the rationale for applying BNCT to tumors that are not responsive to conventional radiation therapy due to their dissemination or infiltrative growth. Since the birth of BNCT, several agents have been developed [2]. Among these, the boron-containing amino acid (L)-4-dihydroxy-borylphenylalanine (^10^B-BPA) [3] and the boron cluster with a mercapto group sodium mercapto-*closo*-undecahydro-dodecaborate (BSH, Na_2_B_12_H_11_SH, sodium borocaptate) are of great clinical interest. (Figure 1) [4].

Today, the most used boron carrier in clinical trials is BPA, which ensures a tumor-to-normal tissue boron concentration ratio of about 3.5:1 [5].

Glioblastoma multiforme (GBM) stands out as one of the most prevalent and aggressive primary brain tumors in adults [6]. Despite standard treatments involving surgical resection and chemoradiotherapy, patient prognosis remains poor, with 2- and 5-year survival rates only reaching 27% and 9.8%, respectively. This underscores the need for alternative therapies, making BNCT a promising option for GBM patients [7]. Realizing the full clinical benefits of BNCT, however, requires the development of highly specific tumor-targeted boronated compounds. Carbohydrate derivatives containing boron are being actively investigated due to the inherent tendency of tumor cells to preferentially accumulate sugars [8]. Among various carbohydrate compounds, glucosamine is notable for its affordability and natural origin. Further, the natural amino sugar N-acetylglucosamine has been specifically identified as an effective targeting agent for malignant cells [9,10].

**Figure 2:**
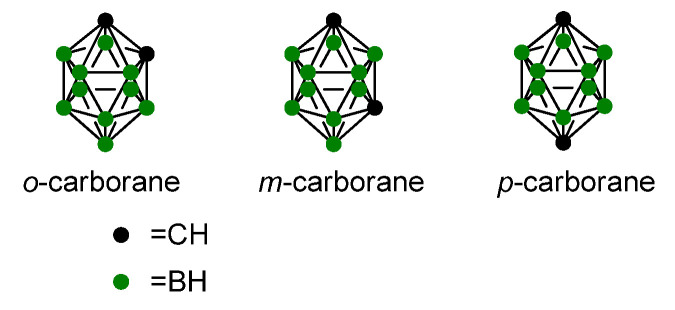
Chemical structures of carboranes.

The entry of *N*-acetylglucosamine (GlcNAc) into cells is stimulated and involves the glucose transporter system. The accumulation is expected in all tumors overexpressing the glucose transporters GLUT-1 and GLUT-2. While GLUT-1 has a similar affinity for glucose and GlcNAc, GLUT-2 showed a twenty-fold higher affinity for GlcNAc than glucose [11]. GLUT-1 is reported to play a significant role in glioma tumor biology, indicating potential for therapeutic effects in patients with malignant gliomas [12]. This work aims to exploit GlcNAc for selectively delivering a boron moiety to tumor cells. We chose the carboranyl unit as the boron-containing feature, given that carboranes are stable boron clusters composed of boron, carbon, and hydrogen atoms (Figure 2). They can conjugate with organic moieties via C-C bonds and have a high boron content, making them ideal for BNCT [13,14]. However, these clusters can have extremely poor solubility in water and can be highly toxic [15]. To overcome this, the conjugation of a polar and highly water-soluble sugar (i.e., GlcNAc) with *o*-carborane led to the final compound **4**, which was sufficiently soluble to be evaluated as a candidate for BNCT treatment.

## 2. Results and Discussion

### 2.1. Synthesis

Over the years, several carborane–carbohydrate conjugates have been proposed as carbohydrate-mediated BNCT agents [16].

**Scheme 1:**
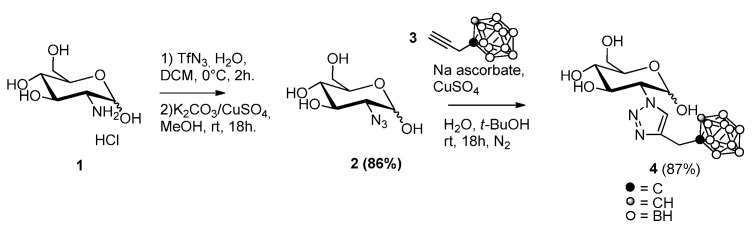
Synthesis of compound **4**.

Commercial glucosamine was first functionalized with an azido group following a reported procedure [16]. For the synthesis of the target compound **4**, an effective approach was used to obtain a wide range of biologically active compounds, namely the Cu(I)-catalyzed azide–alkyne cycloaddition reaction (click chemistry) [17]. Compound **2** was reacted with propargyl-*ortho*-carborane **3** [18] in a mixture of water and t-BuOH with the addition of sodium ascorbate and copper sulphate under a nitrogen atmosphere. The mixture was stirred for 18 h at rt. An improvement in yield over previous attempts was obtained by degassing the solvents before starting the reaction. In detail, before the addition of a solution of copper sulphate, the mixture was put under vacuum for 15 min and then fluxed under a nitrogen atmosphere. The final compound **4** is a mixture of two anomers (α and β), as glucosamine exists as a hemiacetal in equilibrium with its open form when in an aqueous medium. NMR data are provided in the Supporting Information (SI), (Scheme 1).

### 2.2. Solubility

Water solubility is a critical parameter in developing new boronated compounds for BNCT. High doses of these compounds are typically required to achieve therapeutic concentrations via the preferred intravenous administration route [19].

For example, in past clinical applications, sodium borocaptate (BSH) was administered intravenously at a dose of 100 mg/kg body weight 8–14 h before neutron irradiation, followed by an additional dose of 1 mg/kg body weight during irradiation to achieve a boron concentration in blood equal to 30 ppm [20]. Given these stringent requirements for BNCT agents, all new chemical entities (NCEs) for this application undergo a solubility assessment to determine whether intravenous injection is feasible. According to the *European Pharmacopoeia* (*Eur. Ph.* 11th edition), solubility classifications should be carefully considered to avoid biased conclusions.

Despite being approved and used in clinical applications, BPA presents poor water solubility (1.61 ± 0.02 g/L, classified as slightly soluble according to the *Eur. Ph.* 11th edition) [21,22]. However, Yoshino et al. demonstrated that the inclusion of sugars such as fructose significantly enhances water solubility due to the formation of boronate esters with carbohydrate hydroxyl moieties [22]. Therefore, incorporating a carbohydrate moiety into the structure of new potential BNCT agents could be a valuable strategy to develop NCEs with improved intrinsic solubility.

Compound **4** exhibits a poor response with UV-Vis detection; therefore, LC-HRMS was employed to achieve low ppm-level sensitivity and detect small quantities of the dissolved compound. The lower limit of detection (LLOD) under positive Full-MS(+) was 0.100 ppm. In comparison, the lower limit of quantification (LLOQ) was 0.250 ppm (Supplementary Information, Figures S2 and S3). The tandem mass spectrum of **4** is also provided in the Supplementary Materials for its structure elucidation (Figure S1).

The water solubility of **4** at 25 °C was determined to be 4.669 ± 0.366 g/L, classifying it as “slightly soluble” according to the *Eur. Ph.* 11th definition. In conclusion, compound **4** exhibited approximately three-fold higher solubility compared with BPA. Although both compounds are classified as slightly soluble according to the *Pharmacopoeia*, the incorporation of the carbohydrate moiety markedly enhances their intrinsic solubility. Consequently, intravenous administration is enabled, although administration protocol and formulation adjustments may be necessary to achieve effective boron concentrations in malignant tissues.

### 2.3. Cell Viability

**Figure 3:**
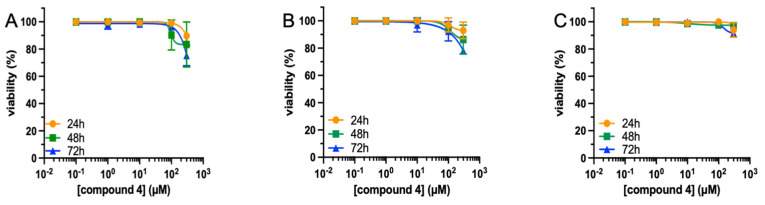
Viability of cell lines exposed to compound **4**. Human U87 MG (**A**), A375 (**B**) cell lines, and human fibroblasts (**C**) were treated with compound 4 (0.1–300 μM) for different times (24–72 h), and cell viability was assessed by MTT assay. Results are the mean ± SEM of three independent experiments, each performed in triplicate.

To evaluate potential cytotoxicity, human primary fibroblasts, A375, and U87-MG cancer cell lines were treated with increasing concentrations (0.1–300 μM) of compound **4**, and cell viability was assessed by MTT assay. As shown in Figure 3, no cytotoxicity was observed until 72 h of treatment. After 72 h, a reduction was observed at the highest concentration (300 μM). The viability measured after 72 h from compound **4** administration was 91.0 ± 1.5%, 78.0 ± 3.4%, and 75.0 ± 1.3% for human fibroblast, A375, and U87-MG cell lines, respectively. These results indicate that compound **4** could be employed for further biological studies at concentrations up to 100 μM. No morphological change was observed for all cell lines at any compound **4** concentration tested (See Figure S4 of SI).

### 2.4. Boron Uptake 

Measurements in control samples (i.e., cells not treated with compound **4**) gave a result comparable with the detection limit (i.e., below 1 ppm), thus demonstrating a lack of boron contamination and ensuring that the boron concentration measured in treated samples was only due to the internalized compound.

**Figure 4:**
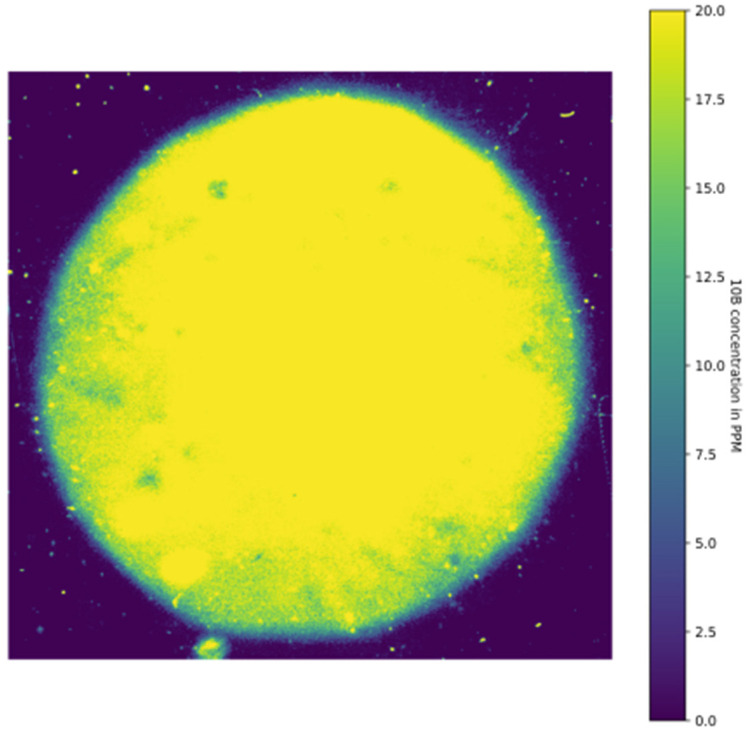
Grayscale uptake measurement for formulation of **4** in one cell pellet previously treated with 38 ppm of boron for 4 h. This pellet corresponds to Sample **4** in Table 1.

Figure 4 shows the result obtained in one of the cell pellets treated at a concentration of 38 ppm of boron. The color map represents boron concentration based on boron imaging obtained by neutron autoradiography. Colors transform the grayscale into a boron concentration value calculated by a calibration curve. Table 1 reports the results for the two pellets for each treatment condition.

The results show that the treatment at the lower concentration is not sufficient, this being the intracellular boron concentration comparable to the control. However, the second protocol was effective in loading tumor cells with a significant ^10^B concentration. It is worth noting that neutron autoradiography can only point out ^10^B isotope, while boron in the preparation was at its natural composition (^11^B 80.1% and ^10^B 19.9%). Thus, a treatment with isotopically enriched compound **4** at 38 ppm would provide around 100 ppm of ^10^B in cells. This encouraging result indicates that compound **4** is taken up by cells in large amounts. The boron concentration of compound **4** provided in the culture medium is 38 ppm (natural isotope), corresponding to a treatment dose of 0.14 mg/mL. The results obtained here can be compared with cell treatment using BPA, where cells are normally exposed to 80 ppm of boron for 4 h, resulting in boron uptake between 20 and 30 ppm, thus achieving a concentration factor (boron concentration obtained in cells/boron concentration provided) of less than 0.5 [23]. Preparation 4 exhibited a concentration factor greater than 2.5, indicating a remarkable capacity to accumulate boron in cells at extremely high concentrations. Although the concentration measured in cells is not representative of boron uptake in vivo, the fact that the presented formulations perform better than BPA could be an indication that it is able to load the tumor with a sufficient boron concentration. For an effective BNCT, it is well established that at least 20 ppm of boron must be achieved in the tumor, considering the typical neutron flux available for clinical treatments [24]. Boronophenylalanine (BPA) can achieve even higher concentrations in GBM, with patient studies reporting levels around 90 ppm [25].

## 3. Materials and Methods

### 3.1. Chemistry

Reagents and solvents were obtained from Sigma-Aldrich (Milan, Italy). Thin-layer chromatography was performed on silica gel plates with a fluorescent indicator (Merck, Milan, Italy) and visualized using UV light (254 nm) and by staining with ceric ammonium molybdate or a 5% sulfuric acid solution in methanol. Flash column chromatography was conducted with 230–400 mesh silica gel (Merck, Milan, Italy). NMR spectra were recorded on a Bruker Avance Neo 400 MHz spectrometer (Bruker, Milan, Italy), with chemical shifts (δ) expressed in parts per million. Coupling constants (J) are reported in Hertz. Mass spectrometry analyses were carried out on a Q-Exactive Plus UHMR Hybrid Quadrupole Orbitrap™ Mass Spectrometer equipped with a Vanquish™ Duo UHPLC system (Waltham, MA, USA). Optical rotations were measured with a JASCO P1010 polarimeter (Jasco Europe, Cremella, Italy) at 20 °C. Infrared spectroscopy was performed using a Bruker Alpha II FTIR (Bruker, Milan, Italy).

(2-(1,2,3-(4-methyl-[1,2-dicarba-*closo*-dodecaboran-1-yl)triazolyl)]-d-glucopyranose) (**4**). In a reaction flask under a nitrogen atmosphere, compounds **2** (135 mg, 0.6 mmol), **3** (100 mg, 0.5 mmol), *t*-butyl alcohol (1.5 mL), sodium ascorbate (87 mg, 0.4 mmol), and water (0.5 mL) were added. The mixture was put under vacuum for 15 min and then stored under a nitrogen atmosphere. A solution of CuSO_4_ (17 mg, 0.4 mmol) in 0.5 mL of water was finally added through a syringe, and the mixture was stirred overnight at room temperature. The reaction was monitored by TLC using as eluent DCM/MeOH in a ratio of 85:15. The solvent was evaporated, and the crude product was purified by flash chromatography (9:1 DCM: MeOH). Yield 87% as a waxy compound. [α]^20^_D_: +38.7 (*c* = 1, methanol). ^1^H NMR: (400 MHz, CD_3_OD anomeric α,β mixture) δ 8.06 (s, 0.5 H, H triazole α), 7.97 (s, 0.5 H, H triazole β), 5.31 (d, *J* = 3.4 Hz, 0.5 H, H1-α), 5.13–5.10 (m, 0.5 H, H1-β), 4.67 (dd, *J* = 11.0, 3.4 Hz, 0.5H, H2-α), 4.41 (bs, 1H), 4.27 (dd, *J* = 11.0, 8.7 Hz, 0.5H, H3-α), 4.17–4.13 (m, 1 H, H2-β and H3-β), 3.99 (d, *J* = 2.4 Hz, 0.5 H, H5-α), 3.95 (dd, *J* = 11.9, 2.0 Hz, 0.5 H, H6a-β), 3.87 (dd, *J* = 11.8, 2.4 Hz, 0.5 H, H6a-α), 3.80 (dd, *J* = 11.9, 4.9 Hz, 0.5 H, H6b-α), 3.77–3.73 (m, 2.5 H, 0.5 H, H6b-β, -CH_2_ carbαβ) 3.58–3.45 (m, 1.5 H, H4-α, H5-β, H4-β), 3.18–1.36 (10H, carb).

^13^C NMR: (101 MHz, CD_3_OD) δ 141.1, 140.8, 125.7 (C triazole β), 123.7 (C triazole α), 94.8 (C1 β), 91.3 (C1 α), 76.8 (C5 β), 74.0 (C4 β, C3 β), 71.9 (C5 α), 71.1 (C4 α), 70.8 (C4 β), 70.3 (C3 α), 68.2 (C4 β, C3 β), 65.4 (C2 α), 61.2 (C6 β), 61.1 (C linker), 61.1 (C6 α), 33.0 (C, triazole-*C*H_2_-carborane). ^11^B NMR: (128 MHz, CD_3_OD) δ −2.5 (d, 1B, *J*_B-H_ = 151.5 Hz), −5.7 (d, 1B, *J*_B-H_ = 147.9 Hz), (−8.8)–(−11.7) (m, 8B). ^11^B dec. NMR: (128 MHz, CD_3_OD) δ −2.5 (s, 1B), −5.7 (s, 1B), (−9.3)–(−11.1) (m, 8B). HRMS (C_11_H_25_B_10_N_3_O_5_; MW 389.2725); found *m*/*z* 390.2781 [M + H]^+^ (vs calcd 390.2803).

### 3.2. Solubility Assessment via LC-MS/MS

*Chemicals and Reagents*. Acetonitrile (ACN), formic acid (FA), and water (LC-MS grade) were purchased from Carlo Erba (Milan, Italy). Compound **4** was synthesized according to the synthesis protocol reported in Section 3.1.

*Protocol*. A saturated water solution of compound **4** was prepared by dissolving 10 mg of powder in 2 mL of water. The centrifuge microtube was gently mixed overnight at 25 °C. After 24 h, the solution was centrifuged, and 1 mL of the supernatant was diluted 20,000-fold using a pre-mixed H_2_O: ACN (7:3, *v*/*v*) solution, matching the initial gradient composition and minimizing the risk of precipitation due to temperature fluctuations. Finally, 1 mL of each duplicate was transferred into LC-MS vials and analyzed.

Calibrators ranging from 0.25 to 10 ppm were prepared in H_2_O: ACN (7:3, *v*/*v*). External calibration was performed using 1/x weighting. Compound **4** concentrations were directly determined using Thermo Fisher’s QuanBrowser Xcalibur software (version 4.1.31.9).

*LC-MS/MS analyses.* LC-MS analyses were conducted using a Q-Exactive Plus UHMR Hybrid Quadrupole Orbitrap Mass Spectrometer interfaced with a Vanquish Duo UHPLC system. Chromatographic separations were achieved on a Polar Synergi column (2.6 µm, 100 Å, 150 × 2.1 mm) kept at 25 °C, protected by a C18 SecurityGuard. A 2.0 µL sample volume was injected, and compounds were eluted at a flow rate of 0.25 mL/min using a mobile phase consisting of (A) 0.1% formic acid in water and (B) 0.1% formic acid in acetonitrile. The gradient elution program was as follows: 30% B for 0.0–1.0 min, increasing to 90% B for 1.0–11.0 min, held at 90% B for 11.0–16.0 min, and returning to 30% B for 16.0–16.2 min, with a total run time of 20.0 min including reconditioning. The autosampler was maintained at 15 °C. An ACN: H_2_O (1:1) solution was used for autosampler needle washes with specific drawing, dispensing, and wash settings. The eluate was introduced into an electrospray ionization (ESI) source. Mass spectrometry data were acquired and processed using Xcalibur software. The Orbitrap mass spectrometer was operated in positive ion mode with a spray voltage of 3.50 kV, a capillary temperature of 300 °C, and an auxiliary gas temperature of 350 °C. Nitrogen was used as sheath gas (45 L/min), sweep gas (1 L/min), and auxiliary gas (10 L/min). The maximum spray current was set to 100 µA, and specific probe positioning settings were applied. Data were acquired both in Full-MS(+) mode (range: 100–2000 *m*/*z*), and in PRM(+) mode at a resolution of 35,000, with an AGC target of 3 × 10^5^, a maximum IT of 120 ms, CE 45, and an isolation window of 4.0 *m*/*z* for the transition MS1: 390.2798 > MS2: 227.2299 *m*/*z*.

### 3.3. Viability Tests

*Cell cultures.* The human primary fibroblasts (ATCC, Accession Numbers PCS-201-012, Lot Number 8221500, American Type Culture Collection, Manassas, VA, USA), U87-MG glioblastoma cell line (ATCC, Accession Numbers: HTB-14), and A375 malignant melanoma cell line (ATCC, Accession Numbers: CRL-1619IG-2) were bought by ATCC. The cell lines were cultured in media as specified by the supplier in a humidified atmosphere (5% CO_2_, 37 °C). Cells were grown in 25 cm^2^ culture flasks and sub-cultured when they reached 80% confluence. The tested compound (4) was dissolved in DMSO to generate the stock solution. The subsequent dilutions were performed in cell culture medium. The maximal DMSO concentration in the cell samples was less than 0.1%.

*Cell viability assay.* Cell lines were seeded (5 × 10^3^ cells/well) in 96-well plates in supplemented media, incubated in a humidified incubator at 37 °C and 5% CO_2,_ and allowed to adhere. After 24 h, cells were treated with increasing concentrations (0.1–300 μM) of compound 4 or DMSO at the same concentrations as in the treated samples for 24–72 h in a humidified incubator at 37 °C and 5% CO_2_. Cell viability was calculated as a percentage using the formula [100(x − y)/(z − y)], where x, y, and z represent the absorbance readings for compound-treated, resting, and compound-untreated cells, respectively. Results are expressed as mean ± S.E.M. of at least three experiments run in triplicate.

### 3.4. Boron Concentration Measurements

U-87 cells were cultured in T-75 flasks in 10 mL of culture medium supplemented with **4** at the concentrations of 3.8 and 38 ppm of boron for 4 h. Subsequently, they were washed three times with Phosphate Buffered Saline (PBS), trypsinized, harvested, and centrifuged to obtain cell pellets, made up of 4 million cells each. Pellets were deposited on circular Mylar disks and allowed to dry overnight. Two pellets were generated for each of the conditions. Control samples were analyzed to verify the absence of boron contamination, thus ensuring reliable results in the treated cells.

Neutron autoradiography uses Solid State Nuclear Track Detectors (SSNTD), passive materials where imprinted images of charged particles can be obtained. Boron present in cell pellets captures thermal neutrons, and the emitted charged particles interact with the SSNTD. The charged particles produce damage along their path, and this damage results in latent tracks. To visualize tracks, a chemical etching is performed by immersion in a suitable solution at fixed temperatures. Such chemical treatment attacks the material with higher velocity in correspondence with the tracks, until they become visible under light microscopy [26]. This technique was set up and calibrated to work in different conditions, and to retrieve information at three different levels: imaging of boron distribution in cell pellets and tissue sections [27], quantitative boron concentration measurements, and sub-cellular boron distribution and concentration evaluation [28]. For this work, we employed the first protocol, which enabled visualization of the boron distribution in shades of gray in the autoradiography images. We then quantified boron using the grayscale and a concurrent calibration with cell pellets of known boron concentration.

Dried pellets were put in contact with the detectors and irradiated in the thermal neutron field inside the thermal column of the TRIGA Mark II reactor of the University of Pavia, Italy (LENA laboratory). The thermal neutron field was fully characterized utilizing experimental measurements and Monte Carlo calculations [29].

Qualitative boron uptake measurements require irradiation of samples and detectors at a high neutron fluence and long etching times. These samples were irradiated at the maximum reactor power of 250 kW for two hours in a position where the neutron flux is about 2·10^9^ cm^−2^ s^−1^. Etching was performed with an NaOH solution at 70 °C for 20 min.

Macroscopic images of the entire sample were obtained using a Leica stereomicroscope; the size of images ranges between 30 and 50 mm^2^, representing boron distribution within the pellet to investigate its uniformity. The qualitative map has thus been converted into colors associated with boron concentration ranges with the Viridis Matplotlib (3.10.3) color map.

## 4. Conclusions

This work describes the synthesis of a novel agent for Boron Neutron Capture Therapy based on a glucosamine structure. The carborane boron carrier was prepared in only two reaction steps, exploiting click chemistry, and obtaining the final product in good yields and high purity. The compound was evaluated for its solubility in water, obtaining a value of 4.669 ± 0.366 g/L. It is considered slightly soluble in the *Eur. Ph.* 11th, allowing intravenous administration. Treatment on human primary fibroblasts, A375, and U87-MG cancer cell lines showed no toxicity up to 100 μM.

Boron uptake was then evaluated on the U87 cell line at this concentration through the autoradiography technique.

Our findings indicate that the novel compound exhibits superior accumulation in tumor cells when compared with the clinically employed Boronophenylalanine (BPA). Specifically, while BPA achieves a boron concentration factor below 0.5, the proposed new formulation demonstrates a factor greater than 2.5. These promising preliminary results suggest improved efficacy in tumor enrichment through systemic administration and warrant further in vivo pharmacokinetic investigations.

## Figures and Tables

**Table 1:** Results of average boron concentration in cell cultures treated with two different protocols. Note that the treatment concentration in the first column refers to boron in natural isotopic composition, while the measurement results in the third column only report the isotope ^10^B.

Sample	Treatment Protocol	^10^B Concentration [ppm]	Uncertainty [ppm]
Control	-	0	1
Sample 1	3.8 ppm 4 h	1	1
Sample 2	3.8 ppm 4 h	1	1
Sample 3	38 ppm 4 h	21.2	0.3
Sample 4	38 ppm 4 h	23.4	0.3

## Data Availability

Data are contained within the article and Supplementary Materials.

## Supplementary Materials

The following supporting information can be downloaded at https://www.mdpi.com/article/10.3390/ph18070986/s1: Figure S1: Mass spectrum of **4**; Figure S2: Compound **4** calibration curve; Figure S3: Compound **4** chromatogram; Figure S4: Cell morphology of human fibroblasts, U87 MG glioblastoma, and A375 melanoma cell lines; Figures S5–S10: NMR Spectra copies of compound **4**.
