# Supplementary material for: A Novel Closo-Ortho-Carborane-Based Glucosamine Derivative as a Promising Agent for Boron Neutron Capture Therapy
Source: Pharmaceuticals (Basel). 2025 Jun 30;18(7):986. doi: 10.3390/ph18070986 (PMC12299632; doi:10.3390/ph18070986)

Supporting Information

A Novel *Closo*-*Ortho*-Carborane-Based Glucosamine Derivative as a Promising Agent for Boron Neutron Capture Therapy

Daniela Imperio[1]*, Ian Postuma[2], Salvatore Villani[3], Erika Del Grosso[3], Laura Cansolino[4,2], Cinzia Ferrari[4,2], Silvia Fallarini[3], Silva Bortolussi[5,2]* and Luigi Panza[3]

1 Department for Sustainable Development and Ecological Transition, University of Eastern Piedmont, Piazza Sant'Eusebio 5, 13100, Vercelli, Italy; daniela.imperio@uniupo.it
2 National Institute of Nuclear Physics (INFN), Unit of Pavia, via A. Bassi 6, 27100, Pavia, Italy; ian.postuma@pv.infn.it
3 Department of Pharmaceutical Sciences, University of Eastern Piedmont, Largo Guido Donegani, 2, 28100, Novara, Italy; salvatore.villani@uniupo.it, erika.delgrosso@uniupo.it, silvia.fallarini@uniupo.it, luigi.panza@uniupo.it,
4 Department of Clinical Surgical Sciences; integrated unit of experimental surgery, advanced microsurgery and regenerative medicine, University of Pavia, via Ferrata 9, 27100, Pavia, Italy;
5 Department of Physics, University of Pavia, via A. Bassi 6, 27100, Pavia, Italy laura.cansolino@unipv.it, cinzia.ferrari@unipv.it, silva.bortolussi@unipv.it,
* Correspondence: daniela.imperio@uniupo.it and silva.bortolussi@unipv.it

a)

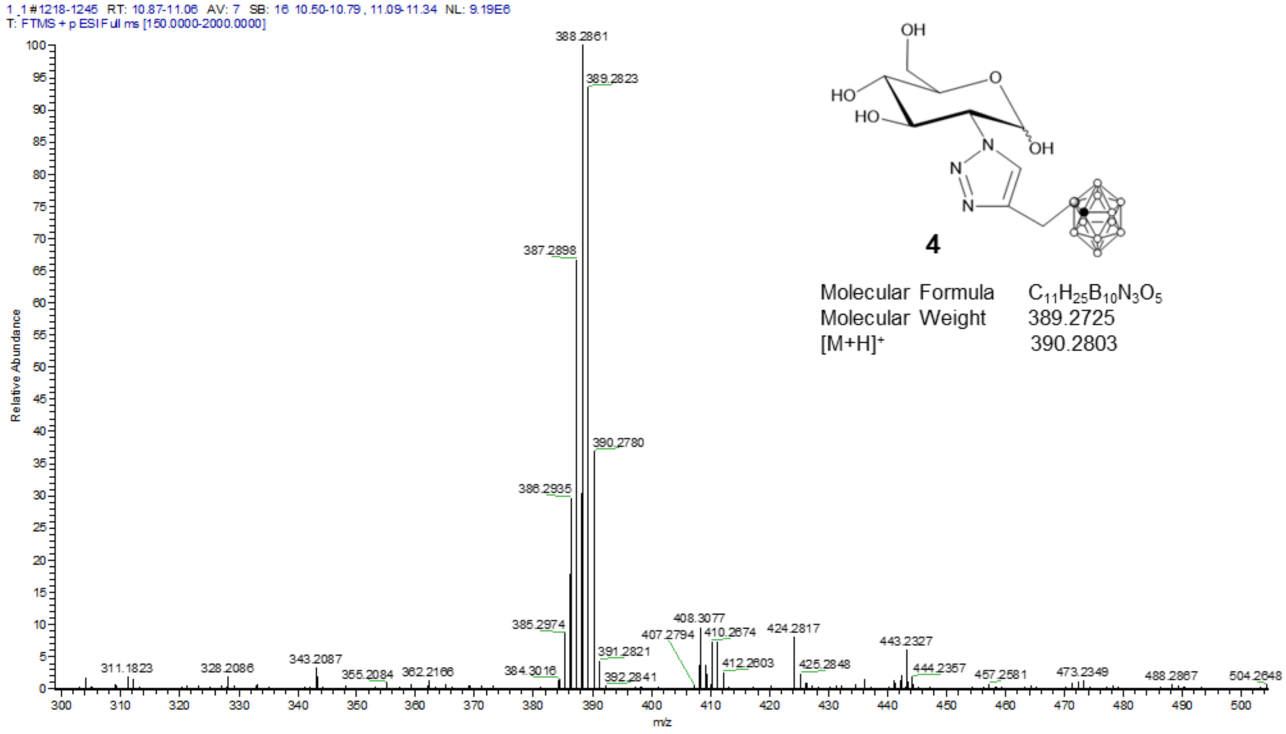
1_1 #1218-1245 RT: 10.87-11.06 AV: 7 SB: 16 10.50-10.79 , 11.09-11.34 NL: 9.19E6
T: FTMS + p ESI Full ms [150.0000-2000.0000]
Relative Abundance
m/z
388.2861
389.2823
387.2898
390.2780
386.2935
385.2974
384.3016
391.2821
392.2841
407.2794
408.3077
410.2674
412.2603
424.2817
425.2848
443.2327
444.2357
457.2581
473.2349
488.2867
504.2648
311.1823
328.2086
343.2087
355.2084
362.2166
4
Molecular Formula C11H25B10N3O5
Molecular Weight 389.2725
[M+H]+ 390.2803

b)

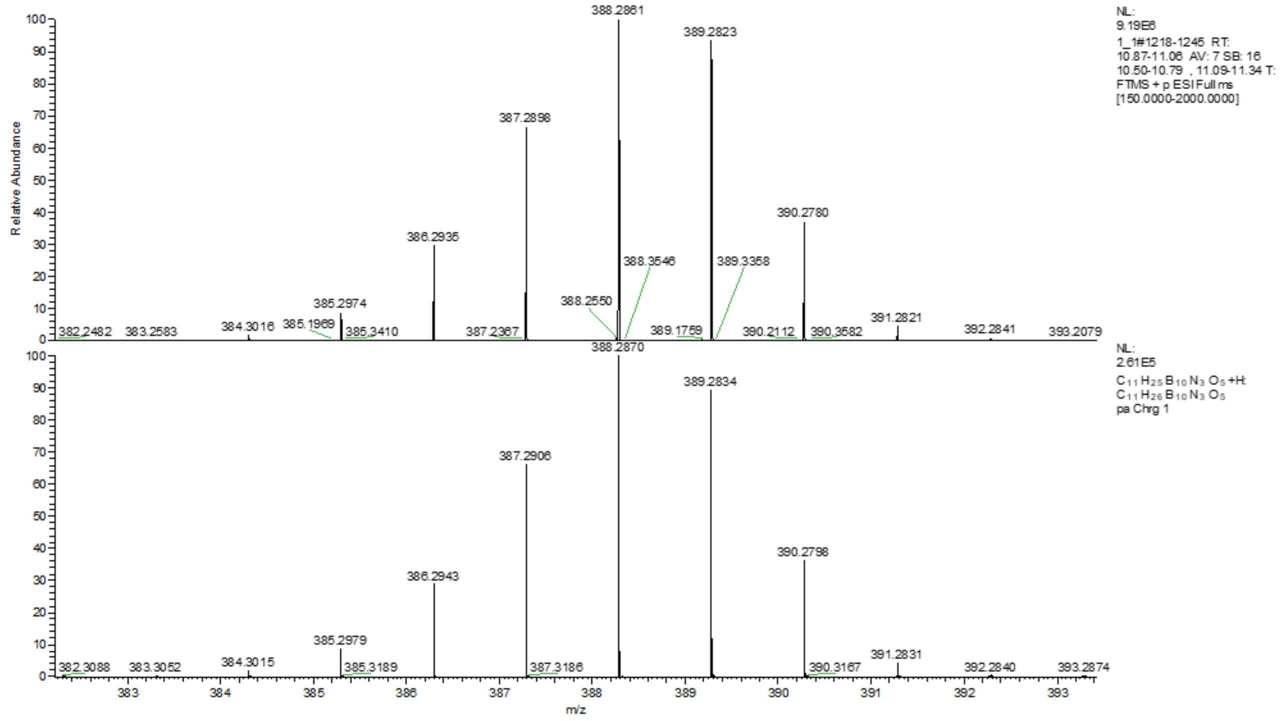
NL:
9.19E6
1_1#1218-1245 RT:
10.87-11.06 AV: 7 SB: 16
10.50-10.79 , 11.09-11.34 T:
FTMS + p ESI Full ms
[150.0000-2000.0000]
NL:
2.61E5
C11 H25 B10 N3 O5 +H:
C11 H26 B10 N3 O5
pa Chrg 1
Relative Abundance
m/z
388.2861
389.2823
387.2898
390.2780
386.2935
385.2974
388.3546
389.3358
388.2550
382.2482
383.2583
384.3016
385.1969
385.3410
387.2367
389.1759
390.2112
390.3582
391.2821
392.2841
393.2079
388.2870
389.2834
387.2906
390.2798
386.2943
385.2979
382.3088
383.3052
384.3015
385.3189
387.3186
390.3167
391.2831
392.2840
393.2874

c)

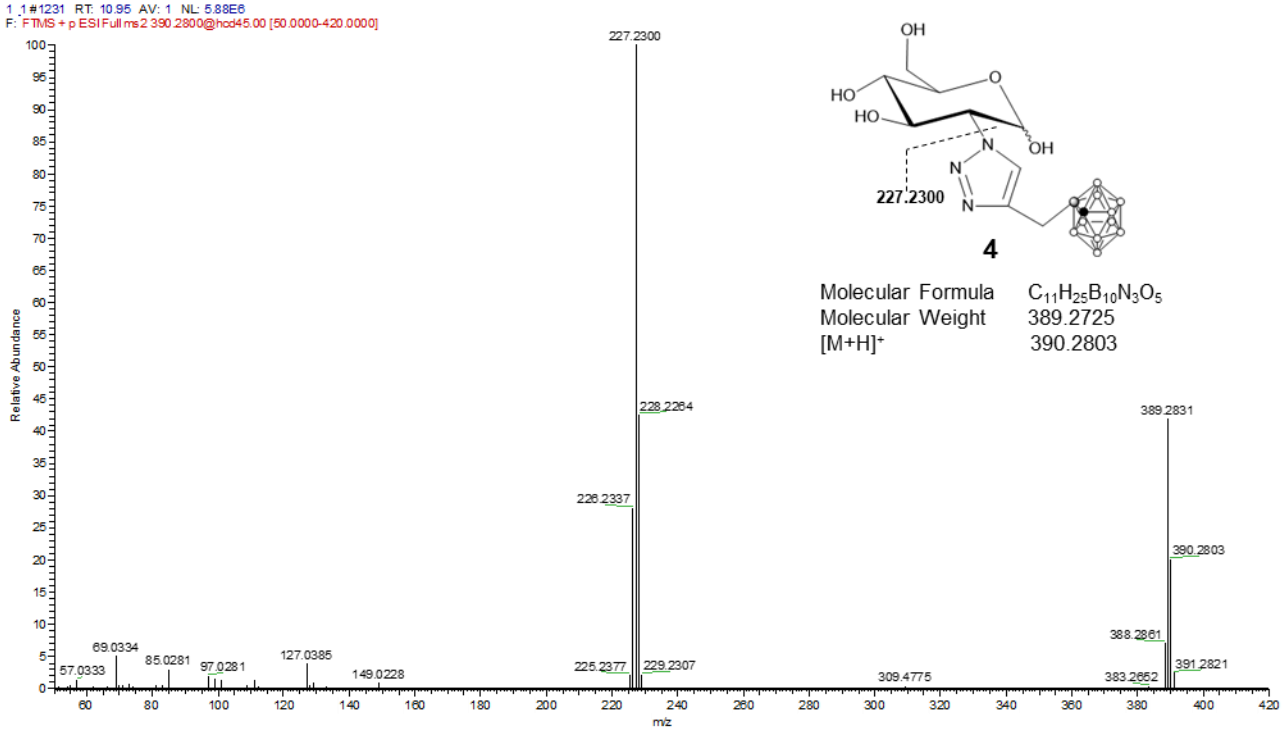

Figure S1. **a)** Full-MS(+), **b)**MS^2 Spectra of **4** and **c)** comparison with calculated mass distribution of the isotopic cluster. To capture the complete isotopic pattern, an isolation window of ±4 m/z was applied, centring on the precursor ion at *m/z* 390.2803. This setting accounts for the natural isotopic distribution of carbon and boron in compound **4**. The base peak observed is the product ion at *m/z* 227.2300, which arises from cleavage of

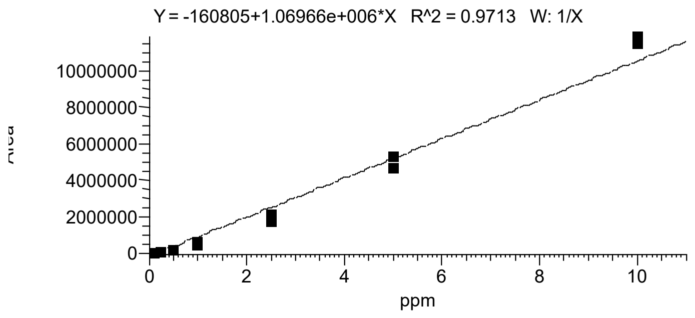

Figure S2. Compound **4** calibration curve in neat H_2O:ACN (7:3, v/v) solvent, ranging from 0.250 to 10 ppm. The LLOD was 0.100 ppm, with no detection at lower concentrations. The coefficient of determination (R^2) was 0.9713.

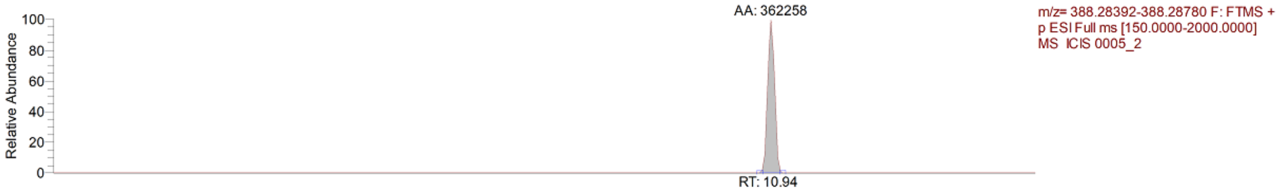

Figure S3. Extracted ion chromatogram (EIC) of compound 4 at 5 ppm, obtained from Full-MS(+) acquisition. The EIC was generated by extracting the base peak at m/z 388.2862 within a 5 ppm mass tolerance.

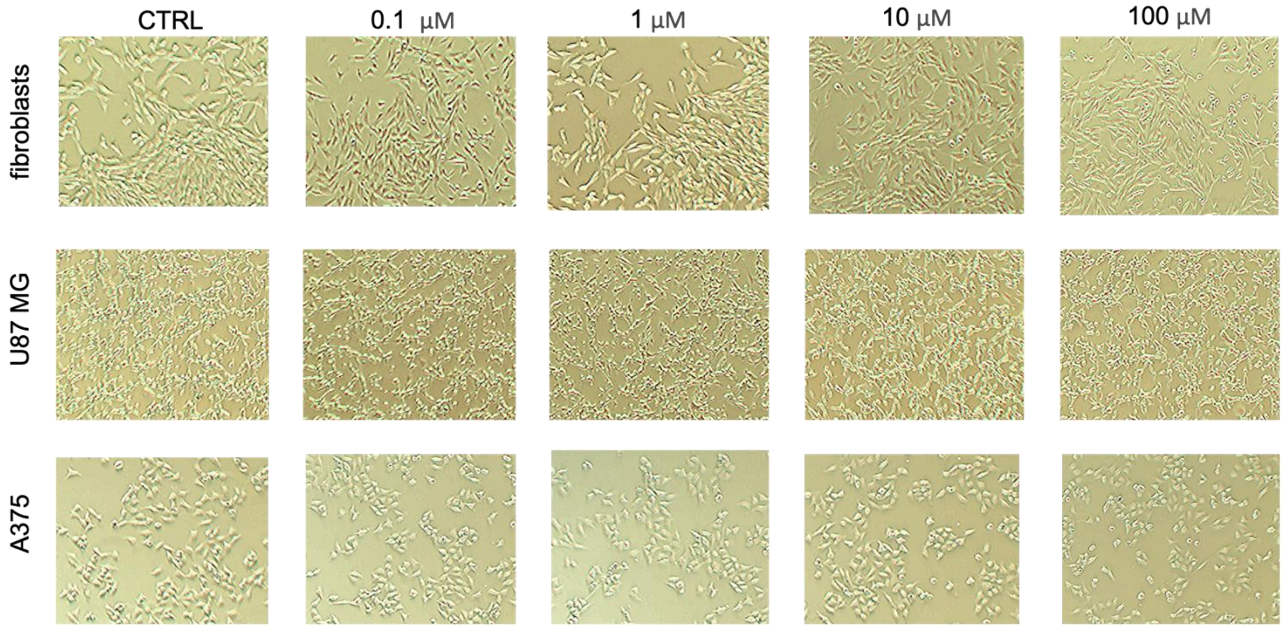

Figure S4. Cell morphology of human fibroblasts, U87 MG glioblastoma, and A375 melanoma cell lines after compound **4** treatment. Bright field Images were taken after 72h of treatment with increasing concentrations of compound **4** with an inverted microscope using a 4X objective. Only images of non-toxic concentration are taken to observe the cell morphology of cell treated/untreated with compound **4**.

Figure S5: NMR ^1H spectrum of compound **4** (400 MHz, CD_3OD anomeric α,β mixture)

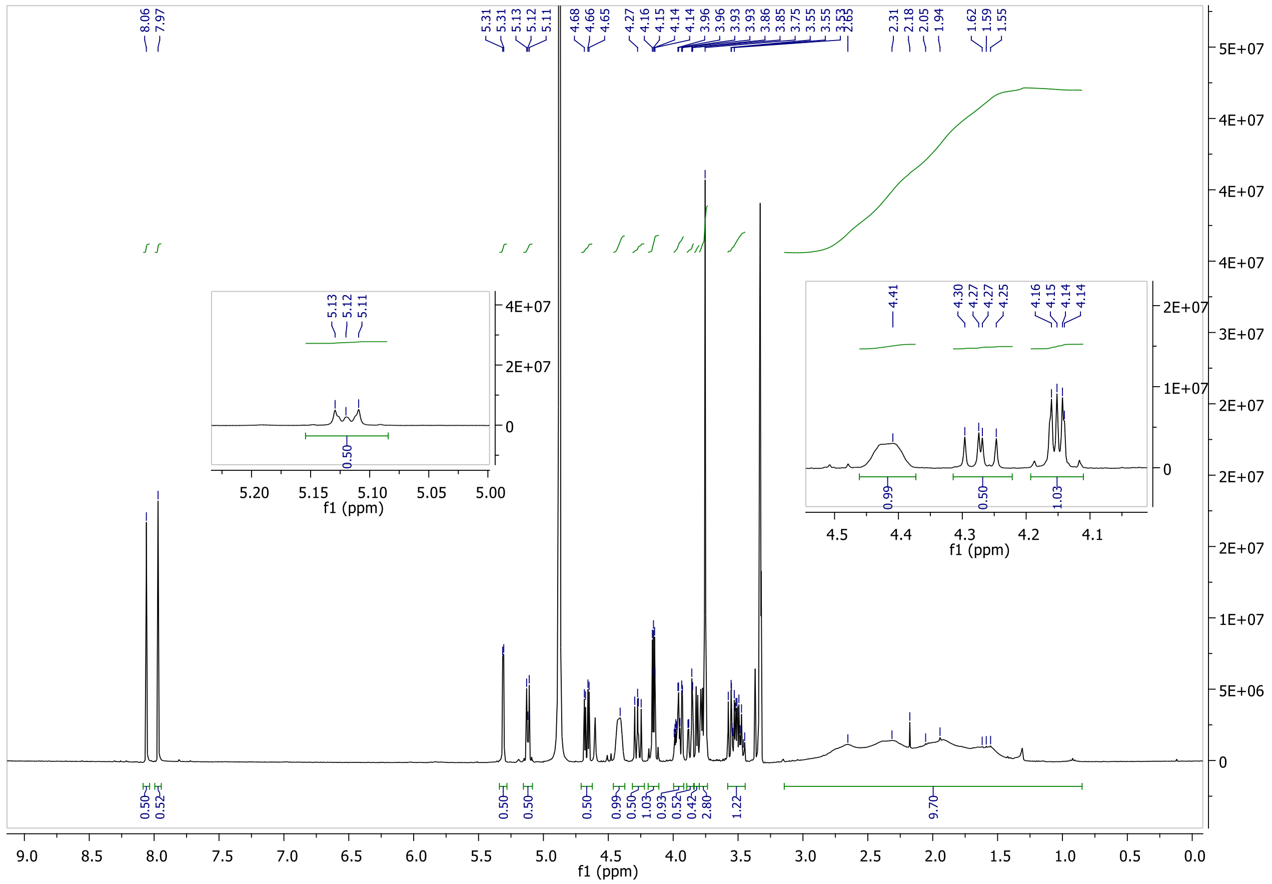

Figure S6: NMR ^{13}C spectrum of compound **4** (101 MHz, CD_3OD)

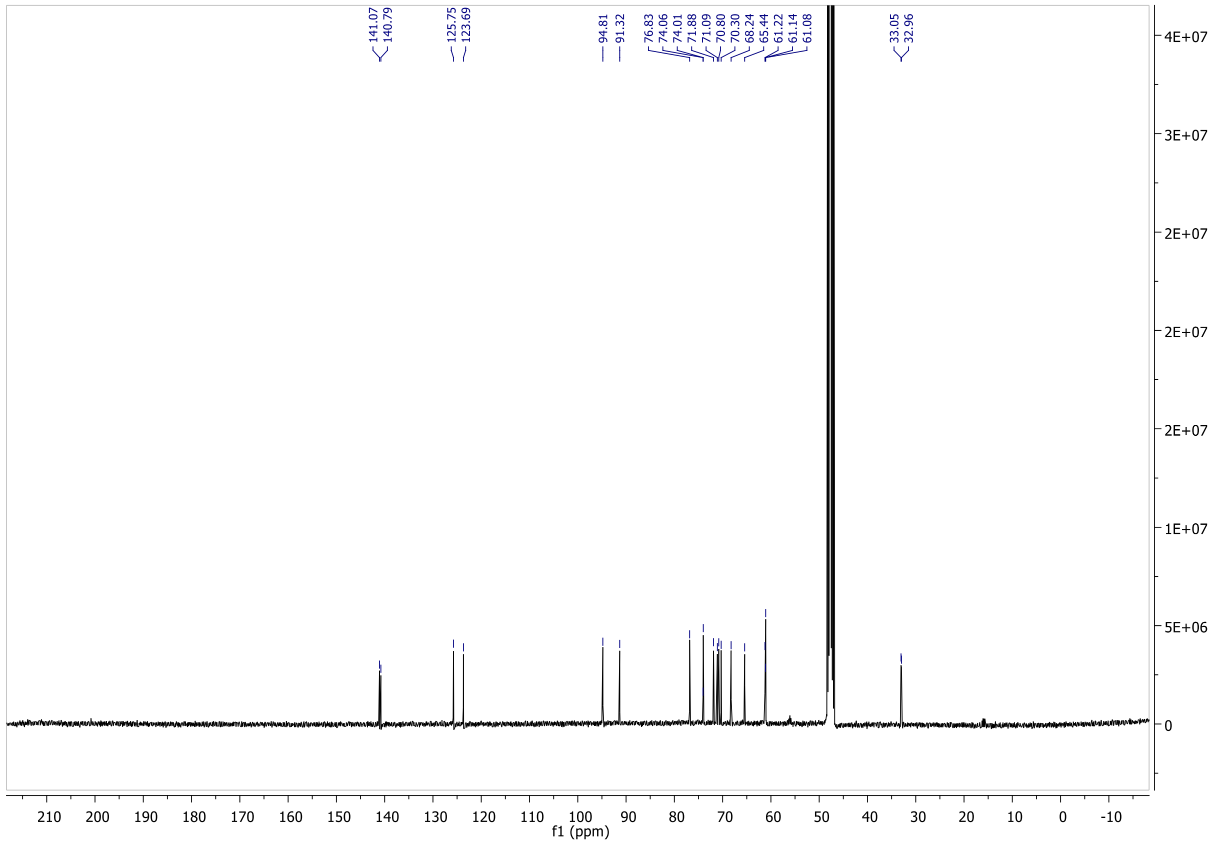

Figure S7: NMR ^{11}B spectrum of compound **4** (128 MHz, CD_3OD)

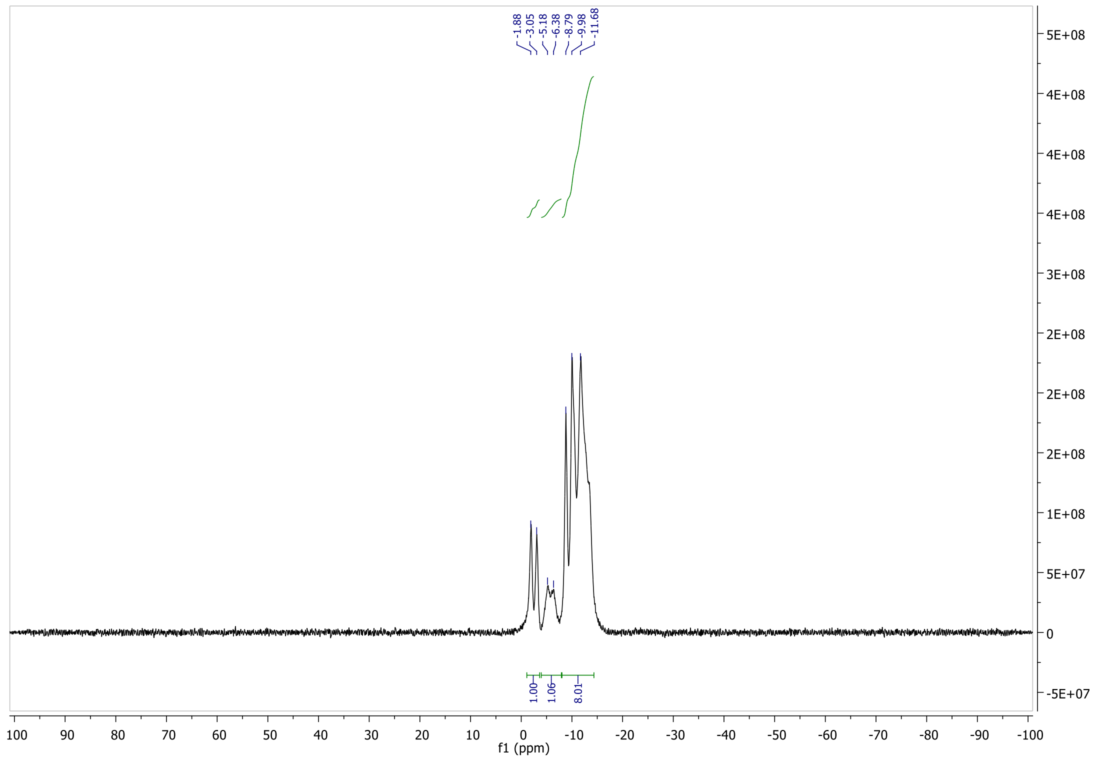

Figure S8: NMR ^{11}B decoupling spectrum of compound **4** (128 MHz, CD_3OD)

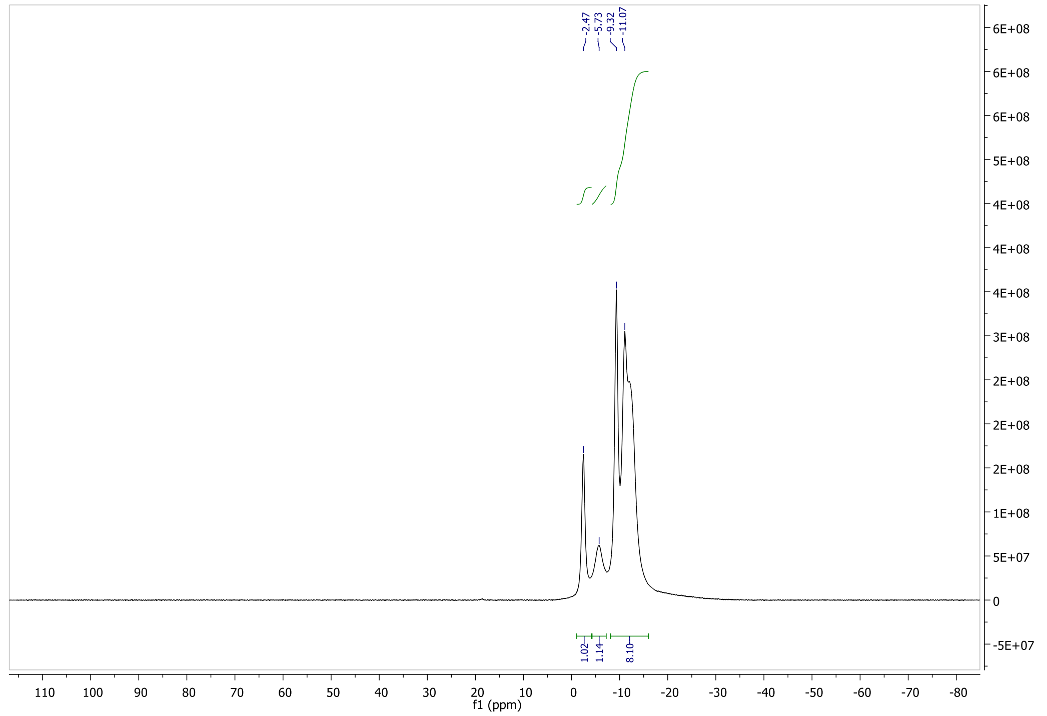

Figure S9: NMR COSY spectrum of compound **4**

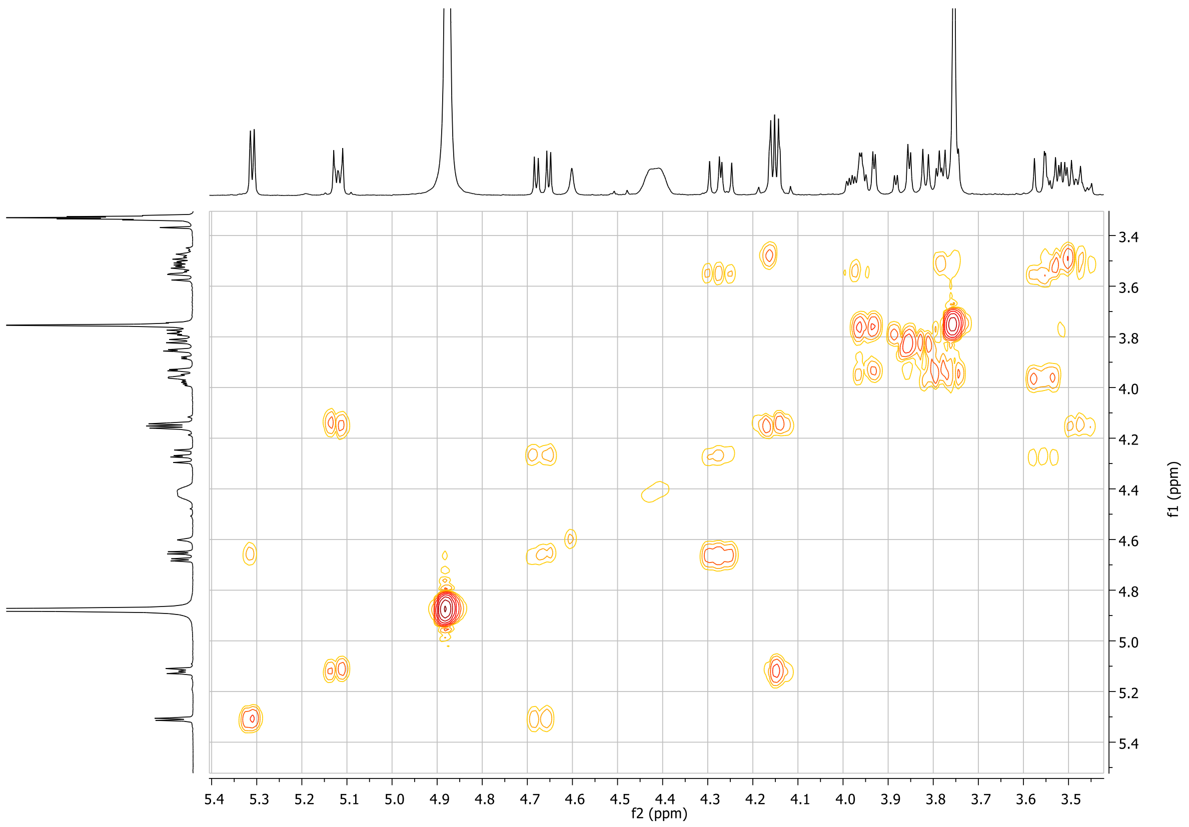

Figure S10: NMR HSQC spectrum of compound **4**

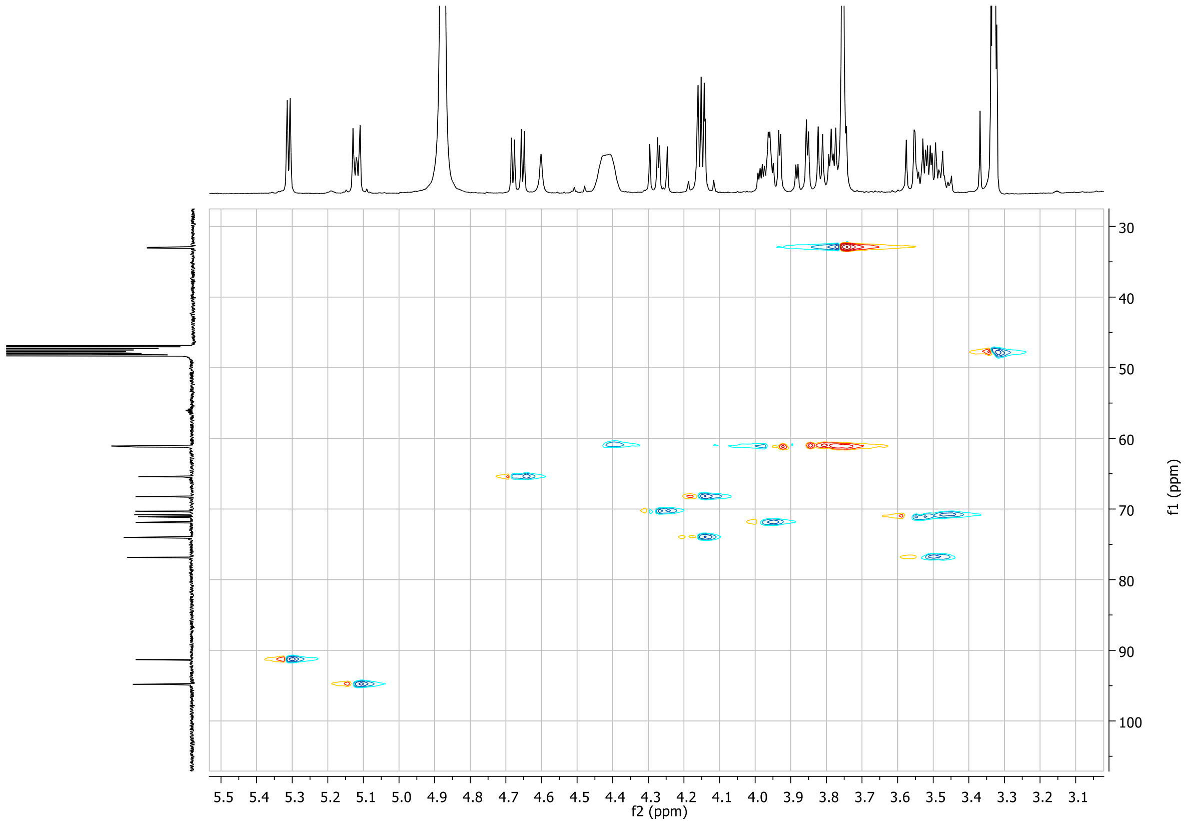

Supplement: Supplementary file 1 [file pharmaceuticals-18-00986-s001.zip › pharmaceuticals-3689726-supplementary.pdf]
